# Root Causing Linearizability Violations

**DOI:** 10.1007/978-3-030-53288-8_17

**Published:** 2020-06-13

**Authors:** Berk Çirisci, Constantin Enea, Azadeh Farzan, Suha Orhun Mutluergil

**Affiliations:** 8 Microsoft Research Lab, Redmond, WA USA; 9 University of Southern California, Los Angeles, CA USA; 10 Université de Paris, CNRS, IRIF, 75013 Paris, France; 11 University of Toronto, Toronto, Canada

## Abstract

Linearizability is the de facto correctness criterion for concurrent data type implementations. Violation of linearizability is witnessed by an error trace in which the outputs of individual operations do not match those of a sequential execution of the same operations. Extensive work has been done in discovering linearizability violations, but little work has been done in trying to provide useful hints to the programmer when a violation is discovered by a tester tool. In this paper, we propose an approach that identifies the root causes of linearizability errors in the form of code blocks whose atomicity is required to restore linearizability. The key insight of this paper is that the problem can be reduced to a simpler algorithmic problem of identifying minimal root causes of conflict serializability violation in an error trace combined with a heuristic for identifying which of these are more likely to be the true root cause of non-linearizability. We propose theoretical results outlining this reduction, and an algorithm to solve the simpler problem. We have implemented our approach and carried out several experiments on realistic concurrent data types demonstrating its efficiency.

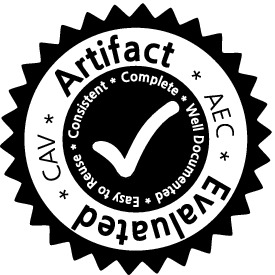



## Introduction

Efficient multithreaded programs typically rely on optimized implementations of common abstract data types (adts) like stacks, queues, sets, and maps [31], whose operations execute in parallel across processor cores to maximize performance [36]. Programming these concurrent objects correctly is tricky. Synchronization between operations must be minimized to reduce response time and increase throughput [23, 36]. Yet this minimal amount of synchronization must also be adequate to ensure that operations behave as if they were executed atomically, one after the other, so that client programs can rely on the (sequential) adt specification; this de-facto correctness criterion is known as *linearizability* [24]. These opposing requirements, along with the general challenge in reasoning about thread interleavings, make concurrent objects a ripe source of insidious programming errors [12, 15, 35].

Program properties like linearizability that are difficult to determine statically are typically substantiated by dynamic techniques like testing and runtime verification. While monitoring linearizability of an execution against an arbitrary adt specification requires exponential time in general [20], there exist several efficient approaches for dealing with this problem that led to practical tools, e.g., [3, 4, 13, 14, 16, 33, 39, 47]. Although these approaches are effective at identifying non-linearizable executions of a given object, they do not provide any hints or guidelines about the source of a non-linearizability error once one is found. If some sort of *root-cause* for non-linearizability can be identified, for example a minimal set of commands in the code that explain the error, then the usability of such testing tools will significantly increase for average programmers. Root-causing concurrency bugs in general is a difficult problem. It is easy enough to fix linearizability if one is willing to disregard or sacrifice performance measures, e.g., by enforcing coarse-grain atomic sections that span a whole method body. It is difficult to localize the problem to a degree that fixing it would not affect the otherwise correct behaviours of the adt. Simplifying techniques, such as equating root causes with some limited set of “bad” patterns, e.g., a non-atomic section formed of two accesses to the same shared variable [10, 28, 38] have been used to provide efficient coarse approximations for root cause identifications.

In this paper, we present an approach for identifying non-linearizability root-causes in a given execution, which equates root causes with optimal repairs that rule out the non-linearizable execution and as few linearizable executions as possible (from a set of linearizable executions given as input). Our approach can be extended to a set of executions and therefore in the limit identify the root cause of the non-linearizability of an adt as a whole. Sequential^1^ executions of a concurrent object are linearizable, and therefore, linearizability bugs can always be ruled out by introducing one atomic section per each method in the adt. Thus, focusing on atomic sections as repairs, there is a guarantee of existence of a repair in all scenarios. We emphasize the fact that our goal is to interpret such repairs as root-causes. Implementing these repairs in the context of a concrete concurrent object using synchronization primitives (eg., locks) is orthogonal and beyond the scope of this paper. Some solutions are proposed in [28, 29, 46].

As a first step, we investigate the problem of finding *all optimal repairs* in the form of sets of atomic sections that rule out a given (non-linearizable) execution. A repair is considered optimal when roughly, it allows a maximal number of interleavings. We identify a connection between this problem and *conflict serializability* [37], an atomicity condition originally introduced in the context of database transactions. In the context of concurrent programs, given a decomposition of the program’s code into code blocks, an execution is conflict serializable if it is equivalent^2^ to an execution in which all code blocks are executed in a sequential non-interleaved fashion. A repair that rules out a non-linearizable execution $\tau$ can be obtained using a decomposition of the set of events in $\tau$ into a set of blocks that we call intervals, such that $\tau$ is *not* conflict serializable with respect to this decomposition. Each interval will correspond to an atomic section in the repair (obtained by mapping events in the execution to statements in the code). A naive approach to compute all optimal repairs would enumerate all decompositions into intervals and check conflict-serializabiliy with respect to each one of them. Such an approach would be inefficient because the number of possible decompositions is exponential in both the number of events in the execution and the number of threads. We show that this problem is actually *polynomial time* assuming a fixed number of threads. This is quite non-trivial and requires a careful examination of the cyclic dependencies in non conflict-serializable executions. Assuming a fixed number of threads is not an obstacle in practice since recent work shows that most linearizability bugs can be caught with client programs with two threads only [12, 15].

**Fig. 1.**
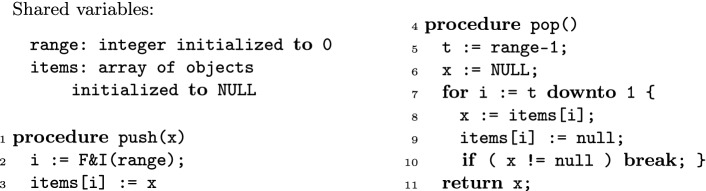
A non-linearizable concurrent stack.

In general, there may exist multiple optimal repairs that rule out a non-linearizable execution. To identify which repairs are more likely to correspond to root-causes, we rely on a given set of *linearizable* executions. We rank the repairs depending on how many linearizable executions they disable, prioritizing those that exclude fewer linearizable executions. This is inspired by the hypothesis that cyclic memory accesses occurring in linearizable executions are harmless.

We evaluated this approach on several concurrent objects, which are variations of lock-based concurrent sets/maps from the Synchrobench repository [21]. We considered a set of non-linearizable implementations obtained by modifying the placement of the lock/unlock primitives, and applied a linearizability testing tool called Violat [14] to obtain client programs that admit non-linearizable executions. We applied our algorithms on the executions obtained by running these clients using Java Pathfinder [44]. Our results show that our approach is highly effective in identifying the precise root cause of linearizability violations since in every case, our tool precisely identifies the root cause of a violation that is discoverable by the client of the library used to produce the error traces.

**Fig. 2.**
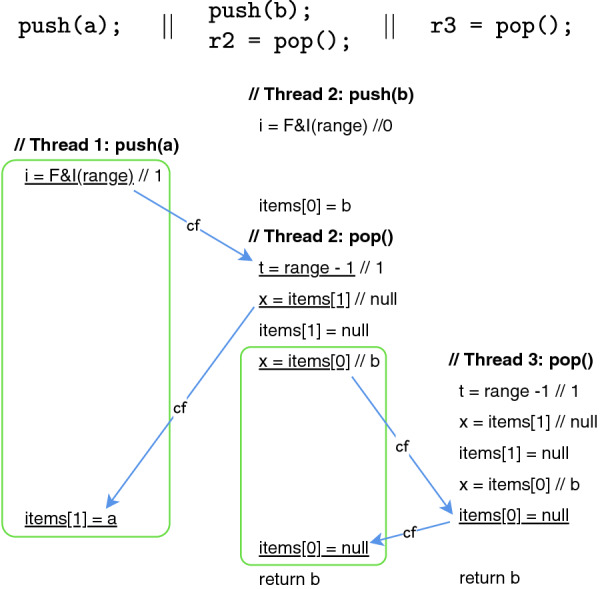
A client program of the concurrent stack of Fig. 1 and one of its non-linearizable executions illustrate as a sequence of read/write events.

## Overview

Figure 1 lists a variation of a concurrent stack introduced by Afek et al. [1]. The values pushed into the stack are stored into an unbounded array items; a shared variable range keeps the index of the first unused position in items. The push method stores the input in the array and it increments range using a call to an atomic fetch and increment (F&I) primitive. This primitive returns the current value of range while also incrementing it at the same time. The pop method reads range and then traverses the array backwards starting from the predecessor of this position, until it finds a position storing a non-null value. It also nullifies all the array cells encountered during this traversal. If it reaches the bottom of the array without finding non-null values, it returns that the stack is empty.

This concurrent stack is *not* linearizable as witnessed by the execution in Fig. 2. This is an execution of a client with three threads executing two push and two pop operations in total. The push in the first thread is interrupted by operations from the other two threads which makes both pop operations return the same value b. The execution is not linearizable because the value b was pushed only once and it cannot be returned by two different pop operations.

The root-cause of this violation is the non-atomicity of the statements at lines 8 and 9 of pop, reading items[i] and updating it to null. The stack is linearizable when the two statements are executed atomically (see [1]).

Our goal is to identify such root-causes. We start with a non-linearizable execution like the one in Fig. 2. The first step is to compute all optimal repairs in the form of atomic sections that disable the non-linearizable execution. There are two such optimal repairs for the execution in Fig. 2: (1) an atomic section containing the statements at lines 8 and 9 in pop (representing the root-cause), and (2) an atomic section that includes the two statements in the push method.

These repairs disable the execution because each pair of statements is interleaved with *conflicting*^3^ memory accesses in that execution. This is illustrated by the boxes and the edges in Fig. 2 labeled by $\mathsf {cf}$: the boxes include these two pairs of statements and the edges emphasize the order between conflicting memory accesses. In Sect. 5, we formalize this by leveraging the notion of conflict serializability. The execution is not conflict-serializable assuming any decomposition of the code in Fig. 1 into a set of code blocks (transactions) such that one of them contains one of these two pairs. These repairs are optimal because they consist of a single atomic section of minimal size (with just two statements). We formalize a generic notion of optimality in Sect. 4 through the introduction of an order relation between repairs, defined as component-wise inclusion of atomic sections and compute the minimal repairs w.r.t. this order.

At the end of the first phase, our approach produces a set of all such optimal (incomparable) repairs. To isolate one as the best candidate, we use a heuristic to rank the optimal repairs. The heuristic relies on the hypothesis that repairs which disable fewer linearizable executions are more likely to represent the best candidate for the true root-cause of a linearizability bug.

For instance, the client in Fig. 2 admits a linearizable execution where the first two threads are interleaved exactly as in Fig. 2 and where the pop in the third thread executes *after* the first two threads finished. This is linearizable because the pop in the third thread returns the value a written by the push in the first thread in items[1] (this is the first non-null array cell starting from the end). Focusing on the two optimal repairs mentioned above, enforcing only the atomic section in the push will disable this linearizable execution. The atomic section in the pop, which permits this execution, is ranked higher to indicate it as the more likely root-cause. This is the expected result for our example.

This ranking scheme can easily be extended to a set of linearizable executions. Given a set of linearizable executions, we rank optimal repairs by keeping track of how many of the linearizable executions each disables.

## Preliminaries

We formalize executions of a concurrent object as sequences of events representing calling or returning from a method invocation (called *operation*), or an access (read or write) to a memory location. Then, we recall the notion of linearizability [24].

We fix arbitrary sets $\mathbb {M}$ and $\mathbb {V}$ of method names and parameter/return values. We fix an arbitrary set $\mathbb {O}$ of operation identifiers, and for given sets $\mathbb {M}$ and $\mathbb {V}$ of methods and values, we fix the sets $C = \{ {o}. call \ {m}({v}) : m \in \mathbb {M}, v \in \mathbb {V}, o\in \mathbb {O}\}$ and $R = \{{o}. ret \ {v} : v \in \mathbb {O}, o\in \mathbb {O}\}$ of *call actions* and *return actions*. Each call action ${o}. call \ {m}({v})$ combines a method $m \in \mathbb {M}$ and value $v \in \mathbb {V}$ with an *operation identifier* $o\in \mathbb {O}$. A return action ${o}. ret \ {v}$ combines an operation identifier $o\in \mathbb {O}$ with a value $v \in \mathbb {V}$. Operation identifiers are used to pair call and return actions. Also, let $\mathbb {L}$ be a set of (shared) memory locations and $A=\{{o}. rd ({x}),{o}. wr ({x}): o\in \mathbb {O}, x\in \mathbb {L}\}$ the set of *read* and *write* actions. The operation identifier of an action *a* is denoted by $\mathsf {op}({a})$.

We fix an arbitrary set $\mathbb {T}$ of thread ids. An *event* is a tuple ${\left\langle {{t},{a}}\right\rangle }$ formed of a thread id $t\in \mathbb {T}$ and an action $a$. A *trace* $\tau$ is a sequence of events satisfying standard well-formedness properties, e.g., the projection of $\tau$ on events of the same thread is a concatenation of sequences formed of a call action, followed by read/write actions with the same operation identifier, and a return action. Also, we assume that every atomic section (block) is interpreted as an *uninterrupted* sequence of events that correspond to the instructions in that atomic section.

We define two relations over the events in a trace $\tau$: the *program order* relation $\mathsf {po}_\tau$ relates any two events $e_1$ and $e_2$ of the same thread such that $e_1$ occurs before $e_2$ in $\tau$, and the *conflict* relation $\mathsf {cf}_\tau$ relates any two events $e_1$ and $e_2$ of different threads that access the same location, at least one of them being a write, such that $e_1$ occurs before $e_2$ in $\tau$. We omit the subscript $\tau$ when the trace is understood from the context.

Two traces $\tau _1$ and $\tau _2$ are called *equivalent*, denoted by $\tau _1\equiv \tau _2$, when $\mathsf {po}_{\tau _1}=\mathsf {po}_{\tau _2}$ and $\mathsf {cf}_{\tau _1}=\mathsf {cf}_{\tau _2}$. They are called $\mathsf {po}$*-equivalent* when only $\mathsf {po}_{\tau _1}=\mathsf {po}_{\tau _2}$.

The projection of a trace $\tau$ over call and return actions is called a *history* and denoted by $h(\tau )$. A history is *sequential* when each call action *c* is immediately followed by a return action *r* with $\mathsf {op}({c})=\mathsf {op}({r})$. A *linearization* of a history $h_1$ is a sequential history $h_2$ that is a permutation of $h_1$ that preserves the order between return and call actions, i.e., a given return action occurs before a given call action in $h_1$ iff the same holds in $h_2$.

A *library* $L$ is a set of traces^4^. A trace $\tau$ of a library $L$ is *linearizable* if $L$ contains some sequential trace whose history is a linearization of $h(\tau )$. A library is *linearizable* if all its traces are linearizable^5^. In the following, since *linearizability* is used as the main correctness criterion, a *bug* is a trace $\tau$ that is not linearizable.

## Linearizability Violations and Their Root Causes

Given a non-linearizable library, our goal is to identify the root cause of non-linearizability in the library code. Let us start by formally describing the state space of all such causes and state some properties of the space that will aid the understanding of our algorithm. First, our focus is on a specific category of causes, namely those that can be removed through the introduction of new atomic code blocks to the library code without any other code changes.

### Definition 1 (Non-linearizability Root Cause)

For a non-linearizable library $L$, the root cause is formally identified by $\mathcal {R}$, a set of atomic blocks $\mathcal {A}$ such that $L$ is linearizable with the addition of blocks from $\mathcal {A}$.

Observe that the set of atomic blocks identified in Definition 1 can conceptually be viewed as blocks of code whose non-atomicity is the *root cause* of non-linearizability and their introduction would *repair* the library. For the rest of this paper, we use the two terminologies interchangeably since for this specific class, the two notions perfectly coincide. The immediate question that comes to mind is whether Definition 1 is general enough. Observe that since linearizability is fundamentally an atomicity type property for individual methods in a library, if every single method of the library is declared atomic at the code level, then the library is trivially linearizable. The only valid executions of the library are the linear (sequential) executions in this case. Therefore,

### Remark 1

Every non-linearizable library can be made linearizable by adding atomic code blocks in $\mathcal {R}$ according to Definition 1.

Since there always is a trivial repair, one is interested in finding a *good* one. The quality of a repair is contingent on the amount of *parallelism* that the addition of the corresponding atomic blocks removes from the executions of an arbitrary client of the library. Generally, it is understood that the fewer the number of introduced atomic blocks and the shorter their length, the more permissive they will be in terms of the parallel executions of a client of this library. This motivates a simple formal subsumption relationship between repairs of a bug. We say an atomic code block *b* subsumes another atomic code block $b'$, denoted as $b \sqsupseteq _cb'$, if and only if $b'$ is contained within *b*.

### Definition 2 (Repair Subsumption)

A repair $\mathcal {R}$ subsumes another repair $\mathcal {R}'$, we write $\mathcal {R}\sqsupseteq _c\mathcal {R}'$ if and only if for all atomic blocks $b' \in \mathcal {R}'$, there exists an atomic block $b \in \mathcal {R}$ such that $b \sqsupseteq _cb'$.

It is easy to see that $\sqsupseteq _c$ is a partial order, and combined with the finite set of all possible program repairs gives rise to the concept of a set of optimal repairs, namely those that do not subsume any other repair. It can be lifted to sets of repairs in the natural way: $\mathbb {R}\sqsupseteq _c\mathbb {R}'$ iff $\forall \mathcal {R}' \in \mathbb {R}', \exists \mathcal {R}\in \mathbb {R}:\ \mathcal {R}\sqsupseteq _c\mathcal {R}'$.

### Remark 2

The set of traces of a library $L$ with a repair $\mathcal {R}$ is a superset of the set of traces of $L$ with the repair $\mathcal {R}'$ if $\mathcal {R}' \sqsupseteq _c\mathcal {R}$.

This means that an optimal repair identification according to Definition 2 should lead to an optimal amount of parallelism in the library repaired by forcing the corresponding code blocks to execute atomically. The goal of our algorithm is to identify such a set of *optimal repairs*.

Now, let us turn our attention to an algorithmic setup to solve this problem. The non-linearizability of a library $L$ is witnessed by a non-empty set of non-linearizable traces *T*. These are the concrete erroneous traces of (a client of) the library, for which we intend to identify the repair.

Note that if $\tau$ is a non-linearizable trace, then all the traces $\tau '$ that are equivalent to $\tau$ are also non-linearizable. Indeed, if $\tau '$ is equivalent to $\tau$, then the values that are read in $\tau '$ are the same as in $\tau$^6^, which implies that the return values in $\tau '$ are the same as in $\tau$, and therefore, $\tau '$ is non-linearizable when $\tau$ is.

Consider a conceptual oracle, $\mathcal {O}^{L}(T)$, that takes a set of non-linearizable traces of a library $L$ and produces the set of all optimal repairs $\mathbb {R}$ such that each $\mathcal {R}\in \mathbb {R}$ excludes all the traces that are equivalent to those in *T*. Then the following iterative algorithm produces $\mathbb {R}$ for a library $L$:

1. Let $$T = \emptyset $$ and $$\mathbb {R}= \emptyset $$.
2. Check if $L$ with the addition of atomic blocks from $\mathbb {R}$ is linearizable:
  Yes? Return $$\mathbb {R}$$.
  NO? Produce a set of non-linearizability witnesses $T'$ and let $T = T \cup T'$.
3. Call $\mathcal {O}^L(T)$ and update the set of repairs $\mathbb {R}$ with the result.
4. Go to back to step 2.

### Proposition 1

The above algorithm produces an optimal set of repairs $\mathbb {R}$ that make its input library linearizable.

It is easy to see that if oracle $\mathcal {O}^L(T)$ can be relied on to produce perfect results, then the algorithm satisfies a progress property in the sense that $\mathbb {R}_{k+1} \sqsupseteq _c\mathbb {R}_k$, where $\mathbb {R}_k$ is the value of $\mathbb {R}$ in the *k*-th iteration of the loop. Following Remark 1, this chain of increasingly stronger repairs is bounded by the specific repair in which every method of the library $L$ has to be declared atomic. Therefore, the algorithm converges. The assumption of optimality for $\mathcal {O}^L(T)$ implies that on the iteration that the algorithm terminates, it will produce the optimal $\mathbb {R}$.

Note that in oracle $\mathcal {O}^L$, the focus shifts from identifying the source of error for the entire library to identifying the source of error in a specific set of non-linearizability witnesses. First, we propose a solution for implementing $\mathcal {O}^L$ for a singleton set, i.e. precisely one error trace, and later argue why the solution easily generalizes to finitely many error traces.

### Repair Oracle Approximation

Given a trace $\tau$ as a violation of linearizability, we wish to implement $\mathcal {O}^L$ that takes a single trace $\tau$ and proposes an optimal set of repairs for it.

Observe that if every trace of $L$ is *conflict serializable* [37] (i.e., equivalent to a sequential trace), assuming method boundaries as transaction boundaries, then it is necessarily linearizable. Therefore, knowing that it is not linearizable, we can conclude that there exists some trace of $L$ which is not serializable. Following the same line of reasoning, we can conclude that the error trace $\tau$ itself is not *conflict serializable*, for some choice of transaction boundaries. This observation is the basis of our solution for approximating repairs for non-linearizability through an oracle that is actively seeking to repair for non-serializability violations.

#### Definition 3 (Trace Eliminator)

For an error trace (a bug) $\tau$, a set of atomic blocks $\mathcal {R}$ is called a *trace eliminator* if and only if every trace that is equivalent to $\tau$ is not a trace of the new library with the addition of blocks from $\mathcal {R}$.

Any trace eliminator that removes $\tau$ as a valid trace of a client of the library $L$ (and all the traces equivalent to $\tau$), by amending the library for the conflict serializability violation, (indirectly) eliminates it as a witness to non-linearizability as well. Note that the universes of *trace eliminators* and *non-linearizability repairs* are the same set of objects, and therefore the subsumption relation $\sqsupseteq _c$ is well defined for trace eliminators, and the concept of optimality is similarly defined. Moreover, Definition 3 is agnostic to linearizability and can be interchangeably used for serializability repairs.

#### Theorem 1

$\mathcal {R}$ is a trace eliminator for $\tau$ if and only if $\tau$ is not conflict serializable with transaction boundaries that subsume $\mathcal {R}$ (statements that are not included in the atomic sections from $\mathcal {R}$ are assumed to form singleton transactions).

#### Proof

(Sketch) For the if direction, assume by contradiction that $\mathcal {R}$ is not a trace eliminator for $\tau$. This implies that there exists a trace $\tau '\equiv \tau$ where the sequences of events corresponding to the atomic sections in $\mathcal {R}$ occur uninterrupted (not interleaved with other events). This is a direct contradiction to $\tau$ not being conflict serializable when transaction boundaries are defined precisely by the atomic sections in $\mathcal {R}$. For the only if direction, assume by contradiction that $\tau$ is conflict serializable. By definition, there is an equivalent trace $\tau '$ where the sequences of events corresponding to the atomic sections in $\mathcal {R}$ occur uninterrupted. Therefore, the library $L'$ obtained by adding the atomic code blocks in $\mathcal {R}$ admits $\tau '$, which contradicts the fact that $\mathcal {R}$ is a trace eliminator for $\tau$.   $\square$

The relationship between the set of trace eliminators for $\tau$ and $\mathcal {O}^L(\tau )$ can be made precise. Since every trace eliminator is a linearizability repair by definition, but not necessarily an optimal one, we have:

#### Proposition 2

Let $\mathcal {O}^L(\tau )$ represent the optimal set of repairs that eliminate $\tau$ as a witness to non-linearizability and $\mathbb {R}$ be the set of optimal trace eliminators for $\tau$. We have $\mathbb {R} \supseteq \mathcal {O}^L(\tau )$.

This is precisely why the set of trace eliminators safely overapproximates the set of linearizability repairs for a single trace. Note that Theorem 1 links any trace eliminator (a set of code blocks) to a collection of dynamic (runtime) transactions. It is fairly straightforward to see that given the latter as an input, the former can be inferred in a way that the dynamic transactions generated by the static code blocks are as close as possible to the input transaction boundaries, assuming no structural changes occur in the code. In Sect. 5, we discuss how an optimal set of dynamic transaction boundaries can be computed, which give rise to a set of optimal trace eliminators.

### Generalization to Multiple Traces

If we have an implementation for an oracle $\mathcal {O}^L(\tau )$ that takes a single trace and produces the set of optimal trace eliminators for it, then the following algorithm implements an oracle for $\mathcal {O}^L(\{\tau _1, \dots , \tau _n\})$ for any finite number of traces:

- Let $$\mathbb {R}= \emptyset $$.
- For each $\tau _i$ ($1 \le i \le n$): let $\mathbb {R}_i = \mathcal {O}^L(\tau _i)$.
- Let $$\mathbb {T} = \mathbb {R}_1 \times \dots \times \mathbb {R}_n$$.
- For each $\mathcal {T} \in \mathbb {T}$: let $\mathbb {R} = \mathbb {R} \cup flatten (\mathcal {T})$.
- For each $\mathcal {R}\in \mathbb {R}$: if $\exists \mathcal {R}' \in \mathbb {R}\text { s.t. } \mathcal {R}\sqsupseteq _c\mathcal {R}'$ then $\mathbb {R}= \mathbb {R}- \{\mathcal {R}\}$.

where $flatten (\mathcal {T})$ basically takes the union of repairs suggested by individual components of $\mathcal {T}$ while merging any overlapping atomic blocks. Note that the *i*th component of $\mathcal {T}$ suggests an *optimal* trace eliminator for $\tau _i$. If we want a tight combination of all such trace eliminators, we need the minimal set of atomic blocks that *covers* all atomic blocks suggested by each eliminator. Formally:

$$ flatten (\langle \mathcal {R}_1, \dots , \mathcal {R}_n\rangle ) = \text { smallest } \mathcal {R}\text { wrt } \sqsupseteq _c\text { st } \forall 1\le i\le n:\ \mathcal {R}\sqsupseteq _c\mathcal {R}_i $$

we can then conclude:

#### Theorem 2

If $\mathcal {O}^L(\tau )$ produces the optimal set of trace eliminators for trace $\tau$, then the above algorithm correctly implements $\mathcal {O}^L(\{\tau _1, \dots , \tau _n\})$, that is, it produces the optimal set of repairs for the set of error traces $\{\tau _1, \dots , \tau _n\}$.

## Conflict-Serializability Repairs

In this section, we investigate the theoretical properties of conflict serializability repairs to provide a set up for an algorithm that implements the oracle $\mathcal {O}^L$ for a single input trace. The goal of this algorithm is to take a trace $\tau$ as an input and return the optimal trace eliminator for $\tau$, under the assumption that $\tau$ witnesses the violation of linearizability.

### Repairs and Conflict Cycles

We start by introducing a few formal definitions and some theoretical connections that will give rise to an algorithm for identifying an optimal set of atomic blocks that can eliminate a trace $\tau$ as a witness to violation of conflict serialiazability.

#### Definition 4 (Decompositions and Intervals)

A *decomposition* of a trace $\tau$ is an equivalence relation $D$ over its set of events such that:

- $D$ relates only events of the same operation, i.e. if $(e_1, e_2) \in D$, then $\mathsf {op}({e_1})=\mathsf {op}({e_2})$, and
- the equivalence classes of $D$ are continuous sequences of events of the same operation, i.e., if $(e_1, e_3) \in D$ and $\{(e_1, e_2), (e_2, e_3)\} \subseteq \mathsf {po}_\tau$, then $\{(e_1, e_2), (e_2, e_3)\} \subseteq D$

The equivalence classes of a decomposition $D$, denoted by $I_{\tau ,D}$ are called *intervals*.

Observe that the relation $\sqsupseteq _c$ is well defined partial order over the universal all possible intervals (of all possible decompositions) of a trace $\tau$.

#### Definition 5 (Interval Graphs)

Given a trace $\tau$, and decomposition $D$, an *interval graph* is defined as $G_{\tau ,D}=(V,E)$ where the set of vertexes *V* is the set of intervals of $D$ and the set of edges *E* is defined as follows

$$\begin{aligned} E = \{(i,i')|\ i \not = i' \wedge \exists e \in i, e' \in i':\ (e,e') \in \mathsf {po}_\tau \cup \mathsf {cf}_\tau \} \end{aligned}$$

Since, by definition, each edge in the interval graph is induced by an edge from either relation $\mathsf {po}_\tau$ or $\mathsf {cf}_\tau$, but note both, we lift these relations over the sets of intervals in the natural way, that is:

$$\begin{aligned} (i,i') \in \mathsf {cf}^i_\tau&\iff \exists e \in i, e' \in i':\ e \ne e' \wedge (e,e') \in \mathsf {cf}_\tau \\ (i,i') \in \mathsf {po}^i_\tau&\iff \exists e \in i, e' \in i':\ e \ne e' \wedge (e,e') \in \mathsf {po}_\tau \end{aligned}$$

Given an interval graph edge $(i,i') \in \mathsf {cf}_\tau ^i \cup \mathsf {po}_\tau ^i$, let

$$\begin{aligned} tre (i,i') = \{(e,e')\ |\ e \in i \wedge e' \in i' \wedge (e,e') \in \mathsf {cf}_\tau \cup \mathsf {po}_\tau \} \end{aligned}$$

**Fig. 3.**
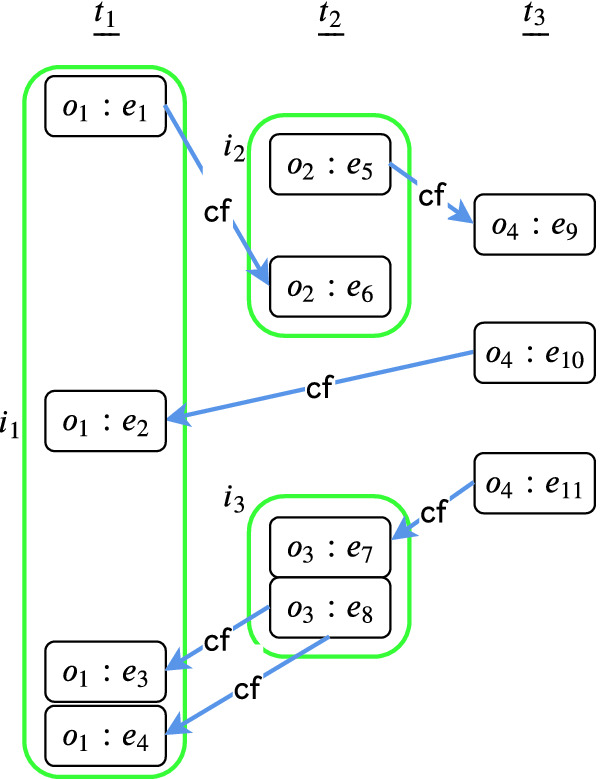
An interval graph.

Figure 3 illustrates an interval graph. Node $o_i:e_j$ denotes an event $e_j$ of operation $o_i$. Events of the same thread are aligned vertically. We draw only $\mathsf {cf}_\tau$ edges since the $\mathsf {po}_\tau$ edges are implied by the vertical alignment of events. Non-singleton intervals of $D$ are $i_1 = \{e_1, e_2, e_3, e_4\}$, $i_2 = \{e_5, e_6\}$ and $i_3 = \{e_7, e_8\}$. Singleton intervals are identified by the corresponding event identifiers. Edges among interval nodes correspond to $\mathsf {cf}_\tau$ or $\mathsf {po}_\tau$. For instance, $(i_1, i_2) \in \mathsf {cf}^i_\tau$ since $(e_1, e_6) \in \mathsf {cf}_\tau$, $e_1 \in i_1$ and $e_6 \in i_2$. As an example for the function $tre$, we have $tre (i_2, i_3) = \{(e_5, e_7), (e_5, e_8), (e_6, e_7), (e_6, e_8) \}$ that consists of $\mathsf {po}_\tau$ edges and $tre (i_3, i_1) = \{(e_8, e_3), (e_8, e_4) \}$ that consists of $\mathsf {cf}_\tau$ edges.

For the degenerate decomposition in which each event is an interval of size one by itself, the interval graph collapses into a *trace graph*, denoted by $G_\tau$. Note that $G_\tau$ is acyclic since the relations $\mathsf {po}_\tau$ and $\mathsf {cf}_\tau$ are consistent with the order between the events in $\tau$.

Intervals are closely related to the static notion of transactions and the induced transaction boundaries on traces. For example, in the decomposition in which the intervals coincide with the boundaries of transactions (e.g. method boundaries), it is straightforward to see that the interval graph becomes precisely the *conflict graph* [19] widely known in the conflict serializability literature. It is a known fact that a trace is conflict serializable if and only if its conflict graph is acyclic [37]. Since $\tau$ is not conflict serializable with respect to the boundaries of methods from $L$, we know the interval graph with those boundaries is cyclic.

With intervals set as single events, $G_\tau$ is acyclic, and with the intervals set at method boundaries, it is cyclic. The high level observation is that there exist a decomposition $D$ in the middle of this spectrum, so to speak, such that $G_{\tau ,D}$ is cyclic, but $G_{\tau ,D'}$ for any $D\sqsupseteq _cD'$ is acyclic. In the following we will formally argue why such a decomposition $D$ is at the centre of identification of serializability repairs.

A cycle in a graph is *simple* if only one vertex is repeated more than once.

#### Definition 6 (Critical Segment Sets)

Let $D$ be a decomposition such that the interval graph $G_{\tau ,D}$ is cyclic and $\alpha = i_0 \dots i_{n-1} i_0$ be a simple cycle. Define

$$\begin{aligned} edges (\alpha )&= tre (i_0, i_1) \times tre (i_1, i_2) \times \dots \times tre (i_{n-1}, i_0)\\ segs (\vec {e})&= \{[e_k^\odot ,e_k^\otimes ]\ |\ 0 \le k \le n-1 \wedge (e_k^\odot , e_{(k+1) \text { mod } n}^\otimes ) = \vec {e}.k \} \\ critSegs (\vec {e})&= \{[e_k^\odot ,e_k^\otimes ] \in segs (\vec {e})\ |\ (e_k^\odot ,e_k^\otimes ) \in \mathsf {po}_\tau \} \\ CritSegs (\alpha )&= \{s\ |\ \exists \vec {e} \in edges (\alpha ):\ s = critSegs (\vec {e})\} \end{aligned}$$

where the set $CritSegs (\alpha )$ is the set of all critical segments sets of cycle $\alpha$.

Note that each cycle may induce several different segment sets, determined by $| edges (\alpha )|$. More importantly, each segment set includes at least one critical segment.

#### Lemma 1

For any $\vec {e} \in edges (\alpha )$, we have $critSegs (\vec {e}) \not = \emptyset$.

#### Example 1

In Fig. 3, $\alpha _1 = i_1, i_2, i_3, i_1$ is a simple cycle. Included in $edges (\alpha )$ are the following three cycles and their corresponding segments:

$$\begin{aligned}&\alpha _1^1 = \langle (e_1, e_6), (e_6, e_7) (e_8, e_3) \rangle&segs (\alpha _1^1) = \{[e_1, e_3], [e_6, e_6], [e_8, e_7] \} \\&\alpha _1^2 = \langle (e_1, e_6), (e_6, e_7), (e_8, e_4) \rangle&segs (\alpha _1^2) = \{[e_1, e_4], [e_6, e_6], [e_8, e_7] \} \\&\alpha _1^3 = \langle (e_1, e_6), (e_5, e_8), (e_8, e_3) \rangle&segs (\alpha _1^3) = \{[e_1, e_3], [e_5, e_6], [e_8, e_8]. \} \end{aligned}$$

The critical segments for these are $critSegs (\alpha _1^1) = \{[e_1, e_3] \}$, $critSegs (\alpha _1^2) = \{[e_1, e_4] \}$ and $critSegs(\alpha _1^3) = \{[e_1, e_3], [e_5, e_6]\}$.

There is a direct connection between the notion of critical segment sets and conflict serializability repairs that the following lemma captures. A segment is called *uninterrupted* in a trace $\tau$ when all its events occur continuously one after another in $\tau$ without an interruption from events of another interval.

#### Lemma 2

Let $\alpha$ be a cycle in some interval graph $G_{\tau ,D}$ of trace $\tau$ which is not conflict serializable wrt to the decomposition $D$ and $critSeg_\alpha \in CritSegs (\alpha )$. There does not exist trace $\tau '$ which is equivalent to $\tau$ in which all segments from $critSeg_\alpha$ are uninterrupted in $\tau '$.

The immediate corollary of Lemma 2 is that if one ensures the atomicity of the segments of events in $CritSegs (\alpha )$ by adding atomic blocks at the code level, then $\tau$ can no longer be an execution of the library. In other words, a set of such atomic code blocks is precisely a trace eliminator (Definition 3) for $\tau$.

### A Simple Algorithm

Lemma 2 and its corollary suggest a simple enumerative algorithm to discover the set of all trace eliminators for a buggy trace $\tau$.

- Let $\mathbb {D}$ be the set of all decompositions of $\tau$ and $\mathbb {R}= \emptyset$.
- For each $$D\in \mathbb {D}$$:
  - Let $\mathbb {C}$ be the set of all simple cycles in $G_{\tau ,D}$.
  - For each $$\alpha \in \mathbb {C}$$:
    - $$*$$ Let $$\mathbb {S} = CritSegs (\alpha )$$.
    - $$*$$ $$\mathbb {R} = \mathbb {R} \cup \mathbb {S}$$
- For each $$\mathcal {R}\in \mathbb {R}$$:
  - If $$\exists \mathcal {R}' \in \mathbb {R}:\ \mathcal {R}\sqsupseteq _c\mathcal {R}'$$ then $$\mathbb {R}= \mathbb {R}- \{\mathcal {R}\}$$.

#### Theorem 3

The above algorithm produces the optimal set of trace eliminators for a buggy trace $\tau$.

This theorem is non-trivial, because the set of cycles considered are limited to simple cycles and an argument is required for why no optimal solution is missed as the result of this limitation. An important point is that any optimal trace eliminator $\mathcal {R}$ defines a decomposition $D$ where the non-singleton intervals are precisely those defined by $\mathcal {R}$ such that $G_{\tau , D}$ contains a simple cycle $\alpha$ and the set of code blocks in $\mathcal {R}$ is a member of $CritSegs (\alpha )$. Note that the algorithm may end up producing non-ideal solutions in the first loop, and the proof of Theorem 3 relies on the argument that all such solutions will be filtered out by a proper solution that guarantees to exist and subsume them.

#### Example 2

The first loop of the above algorithm includes in $\mathbb {R}$ the trace eliminators induced by the critical segments mentioned in Example 1. After the last loop, however, only $critSegs (\alpha _1^1) = \{[e_1, e_3] \}$ will remain in $\mathbb {R}$ since the other two are subsumed by it.

The algorithm is obviously very inefficient. There are two levels of enumeration: all decompositions and all cycles of each decomposition. Assuming that there are $O(|\mathsf {po}_\tau |)$ events in an operation, then there are $O(2^{|\mathsf {po}_\tau }|)$ different decompositions for it. Assuming that there are $O(|\mathbb {T}|)$ operations, we conclude that $|\mathbb {D}| = O(2^{|\mathsf {po}_\tau ||\mathbb {T}|})$. There could be $O(2^{|E_\tau |})$ possible cycles for each decomposition where ${E_\tau = \mathsf {po}_\tau \cup \mathsf {cf}_\tau }$. Therefore, the first loop may generate $O(2^{2|E_\tau ||\mathbb {T}|} )$ many repairs. The last loop iterates over $\mathbb {R}$ and each repair takes $O(\mathbb {R})$ time. The algorithm operates in time $O(2^{4|E_\tau ||\mathbb {T}|} )$. It is exponential both in the size of threads set and the graph. There are many redundancies in the output of the first loop, however. These are exploited to propose an optimized version of this algorithm.

### A Sound Optimization

Consider an arbitrary cycle $\alpha$ in the interval graph $G_{\tau ,D}$. If we want to trace the cycle $\alpha$ over the trace graph $G_\tau$, we would potentially need additional edges that would let us go against the program order inside some intervals that appear on $\alpha$. Let us call the graph extended with such edges $G_\tau ^D$. Formally, $G_\tau ^D$ includes all the nodes and edges from a trace graph and incorporates additional edges between the events of each interval of $D$ to turn it into a *clique*^7^ which is by definition a strongly connected and therefore accommodates the connectivity of any event of an interval to another event in it.

The converse also holds, that is, every *simple* cycle with at least one conflict edge in the $G_\tau ^D$ with the aforementioned additional edges corresponds to a cycle in the interval graph $G_{\tau ,D}$. Note that the inclusion of at least one conflict edge is essential, since every interval graph cycle always includes one such edge by default; since the program order relation is acyclic. Formally:

#### Lemma 3

For each simple cycle $\alpha$ of $G_{\tau ,D}$, there exists a simple cycle $\alpha '$ of $G_\tau ^D$ that contains at most two events from each interval in $\alpha$.

The above lemma can immediately be generalized. Consider the graph $G_\tau ^M$ where *M* indicates the decomposition whose intervals coinciding with the library method boundaries. Since for any arbitrary decomposition $D$, we have $M \sqsupseteq _cD$, we can conclude that $G_\tau ^M$ includes all possible additional edges that one may want to consider as part of a cycle in an arbitrary $G_\tau ^D$ for an arbitrary decomposition $D$. Hence, the set of edges of $G_\tau ^M$ is a superset of the set of edges of all graphs $G_\tau ^D$ for all $D$. This immediately implies that the set of cycles of $G_\tau ^M$ is the superset of the set of cycles of all such graphs. This fact, combined with Lemma 3 leads us to the new simplified algorithm below in place of the one in Sect. 5.2:

- Let $$\mathbb {R}= \emptyset $$.
- Let $\mathbb {C}'$ be the set of all simple cycles in $G_\tau ^M$.
- For each $$\alpha \in \mathbb {C}$$:
  - Let $$\mathbb {S} = critSegs (\alpha )$$.
  - $$\mathbb {R} = \mathbb {R} \cup \mathbb {S}$$
- For each $$\mathcal {R}\in \mathbb {R}$$:
  - If $$\exists \mathcal {R}' \in \mathbb {R}:\ \mathcal {R}\sqsupseteq _c\mathcal {R}'$$ then $$\mathbb {R}= \mathbb {R}- \{\mathcal {R}\}$$.

Note that we are slightly bending the definition of $critSegs$ in the above algorithm, compared to the one given in Definition 6 since the input cycle there is formally a tuple, and here itis simply a list. The function is semantically the same, however and therefore we do not redefine it.

Observe that ever cycle of $G_\tau ^M$ corresponds to a cycle in some graph $G_\tau ^D$ for some decomposition $D$. This observation together with Lemma 3 and Theorem 3 implies the correctness of the above algorithm. Every cycle of every $G_\tau ^D$ is covered by the algorithm, and conversely every cycle considered is valid.

We can simplify the above algorithm one step further by further limiting the set of cycles $\mathbb {C}'$ that need to be enumerated. In graph theory, a chord of a simple cycle is an edge connecting two vertices in the cycle which is not part the cycle.

#### Theorem 4

The above algorithm produces the set of optimal trace eliminators for $\tau$ if $\mathbb {C}'$ is limited to the set of simple chordless cycles of $G_\tau ^M$.

Theorem 4 makes a non-trivial and algorithmically subtle observation. Enumerating the set of all simple chordless cycles of $G_\tau ^D$ is a much simpler algorithmic problem to solve compared to the initial one from Sect. 5.2. Lemma 3 supports part of this argument since it ensures that all repairs explored in the algorithm from Sect. 5.2 are also explored by the above algorithm. For Theorem 4 to hold, one needs to additionally argue that the cycles of $G_\tau ^M$ do not produce any junk, that is, each cycle’s critical segments correspond to a valid trace eliminator for $\tau$. Also, as for simple cycles, $CritSegs (\alpha )$ for a cycle $\alpha$ subsumes $CritSegs (\alpha ')$ for any chordless cycle $\alpha '$ included in $\alpha$. In Sect. 6.1, we present an algorithm that solves the problem of enumerating all cycles in $\mathbb {C}'$ effectively.

## Repair List Generation

In this section, we first start by giving a detailed algorithm that produces the set of all optimal trace eliminators. These repairs suggest incomparable optimal ways of removing an erroneous trace from the library. We then present a novel heuristic that orders this set into a list such that the the ones ranked higher in the list are more likely to correspond to something that a human programmer would identify (amongst the entire set) as the ideal repair.

### Optimal Repairs Enumeration Algorithm

In this section, we present an algorithm for enumerating all simple chordless cycles in $G_\tau ^M$ with at least one $\mathsf {cf}_\tau$ edge, prove its correctness, and formally analyze its time complexity. The algorithm is the following:

- Let $$\mathbb {C}= \emptyset $$.
- For each sequence $\alpha = c_1, c_2,\ldots , c_n$ where $c_i \in \mathsf {cf}_\tau$ and $0 < n \le |\mathbb {T}|$:
  - Let $c_i = (e_i^\otimes , e_i^\odot )$ for all $i \in [1,n]$.
  - If $$(e_i^\odot , e_{(i\ mod\ n) +1}^\otimes ) \in E_\tau ^M\backslash \mathsf {cf}_\tau $$ and $$e_i^\odot \ne e_j^\odot $$ s.t. $$i,j \in [1,n]$$ s.t. $$i \ne j$$:
    - $$*$$ $$\mathbb {C} = \mathbb {C} \cup \{\alpha \} $$

It enumerates all non-empty $\mathsf {cf}_\tau$ sequences of length less than or equal to $|\mathbb {T}|$. If the sequence forms a valid simple cycle and visits each thread at most once (i.e. there are no two distinct conflict edges such that its end points are on the same thread), then it is added to the result set $\mathbb {C}$. Correctness of the algorithm relies on the following observation:

#### Lemma 4

$\alpha$ is a chordless cycle of $G_\tau ^{D}$ with at least one $\mathsf {cf}_\tau$ edge if and only if $\alpha$ visits each thread at most once and it visits at least two threads.

**Fig. 4.**
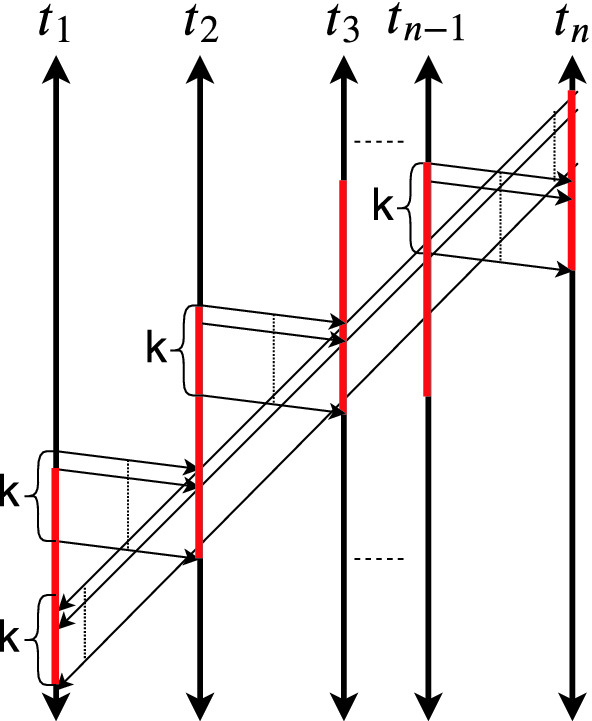
$G_\tau ^M$ with $|\mathsf {cf}_\tau |^{|\mathbb {T}|}$ chordless cycles. (Color figure online)

As a corollary of Lemma 4, we know that a chordless cycle could have at most $|\mathbb {T}|$ conflict edges. Otherwise, by the pigeon hole principle, at least two conflict edges end up in the same thread. Therefore, the algorithm can soundly enumerate only sequences of $\mathsf {cf}_\tau$ edges of length less than or equal to $|\mathbb {T}|$. Moreover, the choice of $\mathsf {cf}_\tau$ determines the rest of the edges in the cycle. Therefore, there are at most $O(|\mathsf {cf}_\tau |^{|\mathbb {T}|})$ chordless cycles with at least one $\mathsf {cf}_\tau$ edge of a graph $G_\tau ^D$.

Note that, in general, the number of simple cycles can be exponential in the number of edges. This means that enumerating only chordless cycles reduces the size asymptotically. In other words, our proposed sound optimization of Sect. 5.3 is at the roof of the polynomial complexity results presented here.

Interestingly, this upper bound is not loose. There is a class of traces parametrized by $|\mathbb {T}|$ such that the number of chordless cycles with at least one $\mathsf {cf}_\tau$ edge is $|\mathsf {cf}_\tau |^{|\mathbb {T}|}$. Let $\mathbb {T}= \{ t_1,\ldots ,t_n\}$ be the set of threads and $G_\tau ^{M}$ has *k* parallel conflict edges between $t_i$ and $t_{(i\ mod\ n) + 1}$ for all $i \in [1,n]$. Moreover, conflict edges that start from $t_i$ is above the conflict edges that end at $t_i$ in terms of program order. This graph is depicted in Fig. 4. To form a cycle, one needs to pick one of *k* edges between $t_i$ and $t_{(i\ mod\ n) + 1}$ for all $i \in [1,n]$. So, there are $k^n$ cycles. Since $k = \frac{|\mathsf {cf}_\tau |}{|\mathbb {T}|}$, there are $\left( \frac{|\mathsf {cf}_\tau |}{|\mathbb {T}|} \right) ^{|\mathbb {T}|}$ chordless cycles with a conflict edge. If we consider $|\mathbb {T}|$ as a constant, there are $\varOmega (|\mathsf {cf}_\tau |^{|\mathbb {T}|})$ chordless cycles with at least one $\mathsf {cf}_\tau$ edge. We are finally ready to state the main complexity result:

#### Theorem 5

Above enumeration algorithm generates all chordless cycles with at least one $\mathsf {cf}_\tau$ edge of $G_\tau ^D$ in $O((|\mathsf {po}_\tau | + |\mathsf {cf}_\tau |) |\mathsf {cf}_\tau |^{|\mathbb {T}|})$ time.

#### Proof

The loop enumerates all the $\mathsf {cf}_\tau$ sequences of length at most $|\mathbb {T}|$ in $O(|\mathsf {cf}_\tau |^{|\mathbb {T}|})$ time. For each such sequence, it takes $O(|\mathsf {po}_\tau | + |\mathsf {cf}_\tau |)$ time to check whether this sequence forms a cycle (if each consecutive conflict edges are connected through a $E_\tau ^M\backslash \mathsf {cf}_\tau$ edge) and whether it visits a thread more than once. As a consequence, the above bound holds.   $\square$

Lastly, there may be as many optimal repairs as there are chordless cycles in $G_\tau ^M$. Consider the class of traces depicted in Fig. 4. Each chordless cycle with at least one $\mathsf {cf}_\tau$ edge has exactly *n* critical segments (illustrated in red). Consider two distinct chordless cycles $\alpha _1$ and $\alpha _2$. There exists a thread $t_i$ such that there is a different edge between $t_i$ and $t_{(i\ mod\ n) + 1}$ in $\alpha _1$ compared to $\alpha _2$. Without loss of generality, assume that the corresponding edge of $\alpha _1$ has source and destination events that appear before the source and destination events of the corresponding edge of $\alpha _2$ in program order ($\mathsf {po}_\tau$). Then, $\alpha _1$ has a larger critical segment on $t_i$ and smaller critical segment in $t_{(i\ mod\ n) + 1}$ compared to $\alpha _2$. Therefore, the neither critical segment subsumes the other. Therefore, each chordless cycle with at least one $\mathsf {cf}_\tau$ edge produces an optimal repair.

This implies that the bound presented in Theorem 5, namely $O((|\mathsf {po}_\tau | + |\mathsf {cf}_\tau |) |\mathsf {cf}_\tau |^{|\mathbb {T}|})$, applies any other algorithm that outputs all optimal repairs.

### Ranking Optimal Repairs

We argued through the example in Sect. 2 and a formal statement in Sect. 4.1 that not every eliminator of a buggy trace $\tau$ is an optimal root cause for non-linearizability. All that we know is that they are all optimal trace eliminators. As a heuristic to identify optimal linearizability repairs out of a set of trace eliminators, we rely on another input in the form of a set $\varGamma$ of linearizable executions, and rank trace eliminators depending on how many linearizable traces from $\varGamma$ they disable, giving preference to trace eliminators that disable fewer ones. This heuristic relies on an experimental hypothesis that there are harmless cyclic dependencies that occur in linearizable executions.

Given a buggy trace $\tau$, and a set $\varGamma$ of linearizable traces, we use the following algorithm to rank trace eliminators for $\tau$:

- Let $\mathbb {R}$ be the set of optimal trace eliminators for $\tau$
- For each $$\mathcal {R}\in \mathbb {R}$$:
  - Let $$f(\mathcal {R})= |\{\tau '\in \varGamma : \mathcal {R}{ isatraceeliminatorfor}\tau '{} \}|$$
- Sort $\mathbb {R}$ in ascending order depending on $f(\mathcal {R})$ with $\mathcal {R}\in \mathbb {R}$.

Since the above algorithm is heuristic in nature, there are no theoretical guarantees for the optimality of its results. For instance, its effectiveness depends on the set of linearizable traces $\varGamma$ given as input. We discuss the empirical aspects of the underlying hypothesis in more detail in Sect. 7.

## Experimental Evaluation

We demonstrate the efficacy of our approach for computing linearizability root-causes on several variations of lock-based concurrent sets/maps from the Synchrobench repository [21]. We consider three libraries from this repository: two linked-list set implementations, with coarse-grain and fine-grain locking, respectively, and a map implementation based on an AVL tree overlapping with two singly-linked lists, and fine-grain locking. We define three non-linearizable variations for each library by shrinking one atomic section only in the add method, only in the remove method, or an atomic section in each of these two methods. For each non-linearizable variation, we use Violat [14] to randomly sample three library clients that admit non-linearizable traces^8^. We use Java Pathfinder [44] to extract all traces of each client, up to partial-order reduction, partitioning them into linearizable and non-linearizable traces. Traces are extracted as sequences of call/return events and read/write accesses to explicit memory addresses, associated to line numbers in the source code of each of the API methods. The latter is important for being able to map critical segments (which refer to events in a trace) to atomic code blocks in the source code.

**Table 1.**
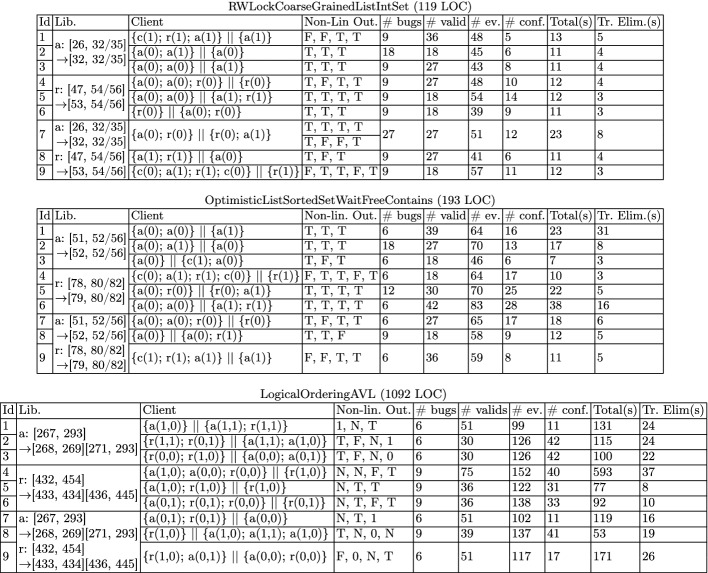
Benchmark data. Column **Lib.** shows the transformation on the atomic section(s) of the original library (we write atomic sections as pairs of line numbers in square brackets), **Client** shows the clients (we abbreviate the names of add, remove, and contains to *a*, *r*, and *c*, resp.), **Non-lin. Out.** shows an outcome (set of return values) witnessing for non-linearizability (true, false, and null are abbreviated to T, F, and N, resp.), **# bugs** and **#valid** give the number of non-linearizable and linearizable traces extracted using Java Pathfinder, respectively, **# ev.** and **# conf.** give the average number of events and conflict edges in these traces, **Total(s)** and **Tr. Elim(s)** give the clock time in seconds for applying our approach, the latter excluding the Java Pathfinder time for extracting traces.

In Table 1, we list some quantitative data about our benchmarks, the clients, and the non-linearizable variations identified by the line numbers of the modified atomic sections (the original libraries can be found in the Synchrobench repository). For instance, the first variation of RWLockCoarseGrainedListIntSet is obtained by shrinking the atomic section in the add method between lines [26, 32/35] to [32, 32/35] (there are two line numbers for the end of the atomic section because it ends with an if conditional).

**Table 2.**
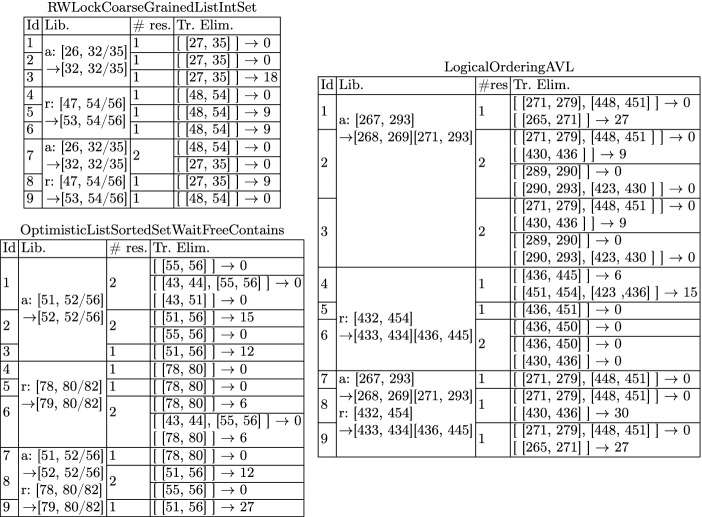
Experimental data. Column **#res** gives the number of different results (sequences of trace eliminators) returned by our algorithm when applied on each of the non-linearizable traces of a client, and **Tr. Elim.** gives the first or the first two trace eliminators in the ranking obtained with our approach. For each trace eliminator we give the number of linearizable traces it disables (after $\rightarrow$).

For each non-linearizable trace $\tau$ of a client *C*, we compute the set of optimal trace eliminators for $\tau$ using the algorithm in Sect. 5.3 with the cycle enumeration described in Sect. 6.1. We then compute the ranking of these trace eliminators using as input the set of linearizable traces of *C* (the restriction to linearizable traces of the same client is only for convenience). Note that multiple trace eliminators can be ranked first since they disable exactly the same number of linearizable traces. Also, note that an optimal root-cause can disable a number of linearizable traces. This is true even for the ground truth repair (i.e. a repair that a human would identify trough manual inspection).

The results are presented in Table 2 and are self-explanatory. In the majority of cases, the first elements in this ranking are atomic sections which are precisely or very close to the expected results, i.e., atomic sections that belong to the original (error-free) version of the corresponding library. In some cases, the output of our approach is close, but not precisely the expected one. This is only due to the particular choice of the client used to generate the traces. In general, the quality of the produced repairs (compared to the ground truth) depends the types of behaviours of the library that the client exercises. However, if our tool ranks repair $\mathcal {R}$ first, in the context of a client *C*, then after repairing the library according to $\mathcal {R}$ the client *C* produces no linearizability violations.

The methods in the libraries OptimisticListSortedSetWaitFreeContains and LogicalOrderingAVL use optimistic concurrency, i.e., unbounded loops that restart when certain interferences are detected. This could potentially guide our heuristic in the wrong direction of giving the ground truth a lower rank. Indeed, a ground truth that concerns statements in the loop body could disable a large number of executions which only differ in the number of loop iterations. This, however, does not happen for small-size clients (like the ones used in our evaluation) since the number of invocations are bounded, which bounds the number of interferences and therefore the number of restarts.

Optimistic concurrency has the potential to mess with the heuristic, but this does not happen in small bounded clients as witnessed by our blah benchmark that does just fine.

To conclude, our empirical study demonstrates that given a good client (one that exercises the problems in the library properly), our approach is very effective in identifying the method at fault and the part of its code that is the root cause of the linearizability violation.

## Related Work

**Linearizability Violations.** There is a large body of work on automatic detection of specific bugs such as data races, atomicity violations, e.g. [18, 40, 41, 45]. The focus of this paper is on linearizability errors. Wing and Gong [47] proposed an exponential-time monitoring algorithm for linearizability, which was later optimized by Lowe [33] and by Horn and Kroening [25]; neither avoided exponential-time asymptotic complexity. Burckhardt et al. [4] and Burnim et al. [5] implement exponential-time monitoring algorithms in their tools for testing of concurrent objects in .net and Java. Emmi and Enea [14, 15] introduce the tool Violat (used in our experiments) for checking linearizability of Java objects.

**Concurrency Errors.** There have been various techniques for fault localization, error explanation, counterexample minimization and bug summarization for sequential programs. We restrict our attention to relevant works for concurrent programs. More relevant to our work are those that try to extract simple explanations (i.e. root causes) from concurrent error traces. In [30], the authors focus on shortening counterexamples in message-passing programs to a set of “crucial events” that are both necessary and sufficient to reach the bug. In [27], the authors introduce a heuristic to simplify concurrent error traces by reducing the number of context-switches. Tools that attempt to minimize the number of context switches, such as SimTrace [26] and Tinertia [27], are orthogonal to the approach presented in this paper. To gain efficiency and robustness, some works rely on simple patterns of bugs for detection and a simple family of matching fixes to remove them, e.g., [10, 28, 29, 38]. Our work is set apart from these works by addressing linearizability (in contrast to simple atomicity violation patterns) as the correctness property of choice, and by being more systematic in the sense that it enumerates all trace eliminators for a given linearizability violation. We also present crisp results for the theoretical guarantees behind our approach and an analysis of the time complexity. Weeratunge et al. [46] use a set of good executions to derive an atomicity “specification”, i.e., pairs of accesses that are atomic, and then enforce it using locks.

There is large body of work on synchronization synthesis [2, 6–8, 11, 22, 34, 42, 43]. The approaches in [11, 42] are based on inferring synchronization by constructing and exploring the entire product graph or tableaux corresponding to a concurrent program. A different group of approaches infer synchronization incrementally from traces [43] or generalizations of bad traces [7, 8]. These techniques [7, 8, 43] also infer atomic sections but they do not focus on linearizability as the underlying correctness property but rather on assertion local violations. Several works investigate the problem of deriving an optimal lock placement given as input a program annotated with atomic sections, e.g., [9, 17, 48]. Afix [28] and ConcurrencySwapper [7] automatically fix concurrency-related errors. The latter uses error invariants to generalize a linear error trace to a partially ordered trace, which is then used to synthesize a fix.

**Linearizability Repairs.** Flint [32] is the only approach we know of that focuses on repairing non-linearizable libraries, but it has a very specific focus, namely fixing linearizability of composed map operations. It uses a different approach based on enumeration-based synthesis and it does not rely on concrete linearizability bugs.

## Footnotes

1 An execution is called sequential when methods execute in isolation, one after another.

2 Two executions are equivalent if roughly, they are the same modulo reordering statements that do not access the same shared variable.

3 As usual, two memory accesses are conflicting when they access the same variable and at least one of them is a write.

4 Intuitively, this corresponds to running a concrete library under a most general client that makes an arbitrary number of invocations from an arbitrary number of threads.

5 Linearizability is typically defined with respect to a sequential ADT. Here, we take the simplifying assumption that the ADT is defined by the set of sequential histories of the library. This holds for all concurrent libraries that we are aware of.

6 We assume that program instructions are deterministic, which is usually the case.

7 A clique is a complete subgraph of a given graph.

8 These linearizability violations are quite rare. The frequencies reported by Violat in the context of a fixed client (when using standard testing) are in the order of 1/1000.
